# Origins of DNA replication

**DOI:** 10.1371/journal.pgen.1008320

**Published:** 2019-09-12

**Authors:** Babatunde Ekundayo, Franziska Bleichert

**Affiliations:** Quantitative Biology, Friedrich Miescher Institute for Biomedical Research, Basel, Switzerland

## Abstract

In all kingdoms of life, DNA is used to encode hereditary information. Propagation of the genetic material between generations requires timely and accurate duplication of DNA by semiconservative replication prior to cell division to ensure each daughter cell receives the full complement of chromosomes. DNA synthesis of daughter strands starts at discrete sites, termed replication origins, and proceeds in a bidirectional manner until all genomic DNA is replicated. Despite the fundamental nature of these events, organisms have evolved surprisingly divergent strategies that control replication onset. Here, we discuss commonalities and differences in replication origin organization and recognition in the three domains of life.

## Introduction

In the second half of the 19th century, Gregor Mendel's pioneering work on the inheritance of traits in pea plants suggested that specific “factors” (today established as genes) are responsible for transferring organismal traits between generations [1]. Although proteins were initially assumed to serve as the hereditary material, Avery, MacLeod and McCarty established a century later DNA, which had been discovered by Friedrich Miescher, as the carrier of genetic information [2]. These findings paved the way for research uncovering the chemical nature of DNA and the rules for encoding genetic information, and ultimately led to the proposal of the double-helical structure of DNA by Watson and Crick [3]. This three-dimensional model of DNA illuminated potential mechanisms by which the genetic information could be copied in a semiconservative manner prior to cell division, a hypothesis that was later experimentally supported by Meselson and Stahl using isotope incorporation to distinguish parental from newly synthesized DNA [4][5]. The subsequent isolation of DNA polymerases, the enzymes that catalyze the synthesis of new DNA strands, by Kornberg and colleagues pioneered the identification of many different components of the biological DNA replication machinery, first in the bacterial model organism *E*. *coli*, but later also in eukaryotic life forms [6].

A key prerequisite for DNA replication is that it must occur with extremely high fidelity and efficiency exactly once per cell cycle to prevent the accumulation of genetic alterations with potentially deleterious consequences for cell survival and organismal viability [7]. Incomplete, erroneous, or untimely DNA replication events can give rise to mutations, chromosomal polyploidy or aneuploidy, and gene copy number variations, each of which in turn can lead to diseases, including cancer [8][9]. To ensure complete and accurate duplication of the entire genome and the correct flow of genetic information to progeny cells, all DNA replication events are not only tightly regulated with cell cycle cues but are also coordinated with other cellular events such as transcription and DNA repair [10][11][12].

**Fig 1:**
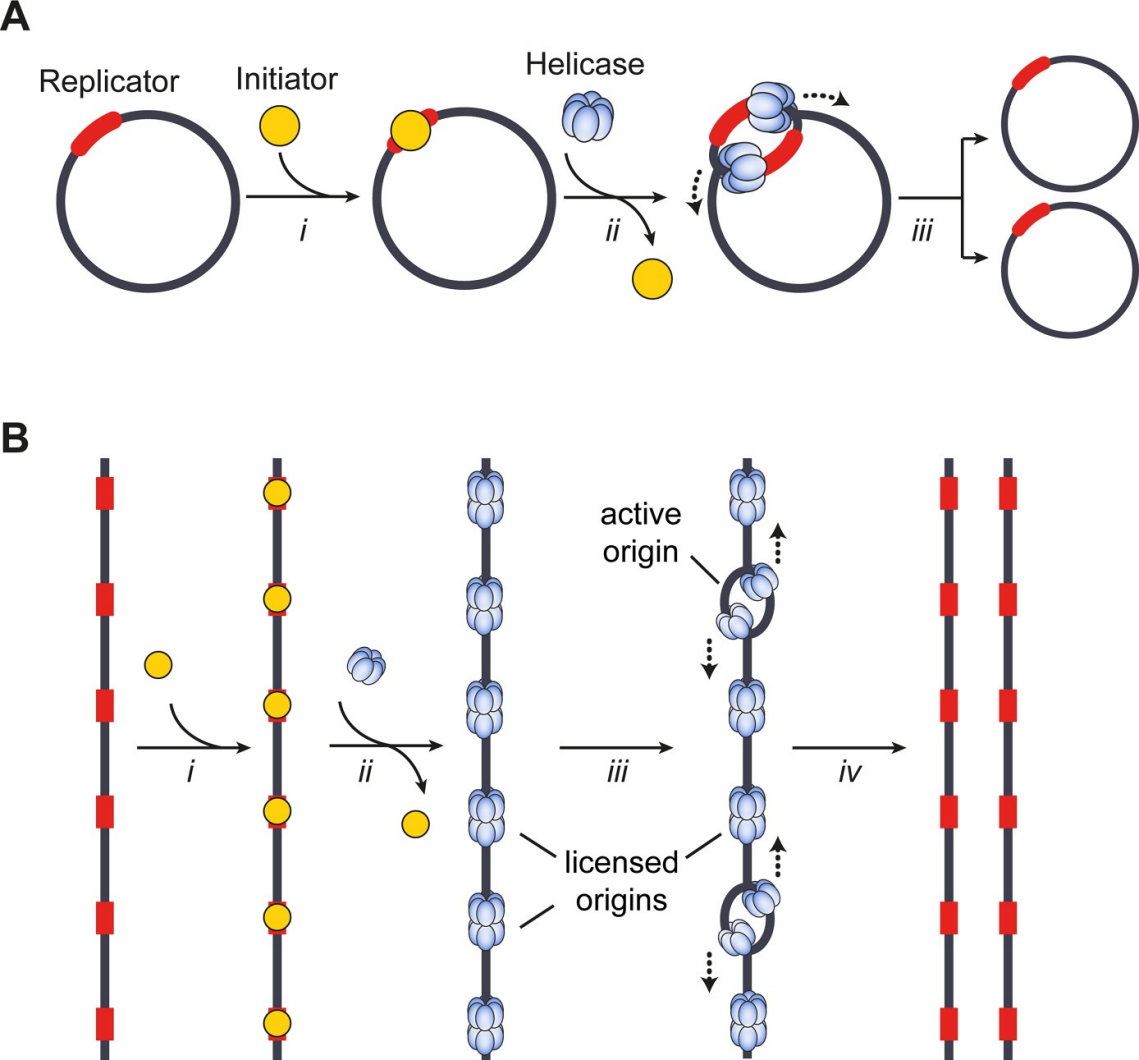
Models for bacterial (**A**) and eukaryotic (**B**) DNA replication initiation. **A**) Circular bacterial chromosomes contain a *cis*-acting element, the replicator, that is located at or near replication origins. *i*) The replicator recruits initiator proteins in a DNA sequence-specific manner, which results in melting of the DNA helix and loading of the replicative helicase onto each of the single DNA strands (*ii*). *iii*) Assembled replisomes bidirectionally replicate DNA to yield two copies of the bacterial chromosome. **B**) Linear eukaryotic chromosomes contain many replication origins. Initiator binding (*i*) facilitates replicative helicase loading (*ii*) onto duplex DNA to license origins. *iii*) A subset of loaded helicases is activated for replisome assembly. Replication proceeds bidirectionally from origins and terminates when replication forks from adjacent active origins meet (*iv*).

DNA replication is divided into different stages (**Fig 1**). During initiation, the replication machineries–termed replisomes–are assembled on DNA in a bidirectional fashion. These assembly loci constitute the start sites of DNA replication or replication origins. In the elongation phase, replisomes travel in opposite directions with the replication forks, unwinding the DNA helix and synthesizing complementary daughter DNA strands using both parental strands as templates. Once replication is complete, specific termination events lead to the disassembly of replisomes. As long as the entire genome is duplicated before cell division, one might assume that the location of replication start sites does not matter; yet, it has been shown that many organisms use preferred genomic regions as origins [13][14]. The necessity to regulate origin location likely arises from the need to coordinate DNA replication with other processes that act on the shared chromatin template to avoid DNA strand breaks and DNA damage [8][12][15][16][17][18][19].

## The replicon model

More than five decades ago, Jacob, Brenner, and Cuzin proposed the replicon hypothesis to explain the regulation of chromosomal DNA synthesis in *E*. *coli* [20]. The model postulates that a diffusible, *trans*-acting factor, a so-called initiator, interacts with a *cis*-acting DNA element, the replicator, to promote replication onset at a nearby origin (**Fig 1A, *i***). Once bound to replicators, initiators (often with the help of co-loader proteins) deposit replicative helicases onto DNA, which subsequently drive the recruitment of additional replisome components and the assembly of the entire replication machinery (**Fig 1A, *ii***). The replicator thereby specifies the location of replication initiation events, and the chromosome region that is replicated from a single origin or initiation event is defined as the replicon.

A fundamental feature of the replicon hypothesis is that it relies on positive regulation to control DNA replication onset, which can explain many experimental observations in bacterial and phage systems [20]. For example, it accounts for the failure of extrachromosomal DNAs without origins to replicate when introduced into host cells. It further rationalizes plasmid incompatibilities in *E*. *coli*, where certain plasmids destabilize each other’s inheritance due to competition for the same molecular initiation machinery [21]. By contrast, a model of negative regulation (analogous to the replicon-operator model for transcription) fails to explain the above findings [20]. Nonetheless, research subsequent to Jacob’s, Brenner’s and Cuzin’s proposal of the replicon model has discovered many additional layers of replication control in bacteria and eukaryotes that comprise both positive and negative regulatory elements, highlighting both the complexity and the importance of restricting DNA replication temporally and spatially [22][23][24].

The concept of the replicator as a genetic entity has proven very useful in the quest to identify replicator DNA sequences and initiator proteins in prokaryotes, and to some extent also in eukaryotes, although the organization and complexity of replicators differ considerably between the domains of life (for reviews, see [25][26]). While bacterial genomes typically contain a single replicator that is specified by consensus DNA sequence elements and that controls replication of the entire chromosome (**Fig 1A**), most eukaryotic replicators–with the exception of budding yeast–are not defined at the level of DNA sequence; instead, they appear to be specified combinatorially by local DNA structural and chromatin cues [27][28][29][30] [31][32][33][34][35][36]. Eukaryotic chromosomes are also much larger than their bacterial counterparts, raising the need for initiating DNA synthesis from many origins simultaneously to ensure timely replication of the entire genome (**Fig 1B**). Additionally, many more replicative helicases are loaded than activated to initiate replication in a given cell cycle (**Fig 1B**). The context-driven definition of replicators and selection of origins suggests a relaxed replicon model in eukaryotic systems that allows for flexibility in the DNA replication program [25]. Although replicators and origins can be spaced physically apart on chromosomes, they often co-localize or are located in close proximity; for simplicity, we will thus refer to both elements as ‘origins’ throughout this review. Taken together, the discovery and isolation of origin sequences in various organisms represents a significant milestone towards gaining mechanistic understanding of replication initiation. In addition, these accomplishments had profound biotechnological implications for the development of shuttle vectors that can be propagated in bacterial, yeast, and mammalian cells [37][38][39].

## Bacterial replication origins

**Fig 2:**
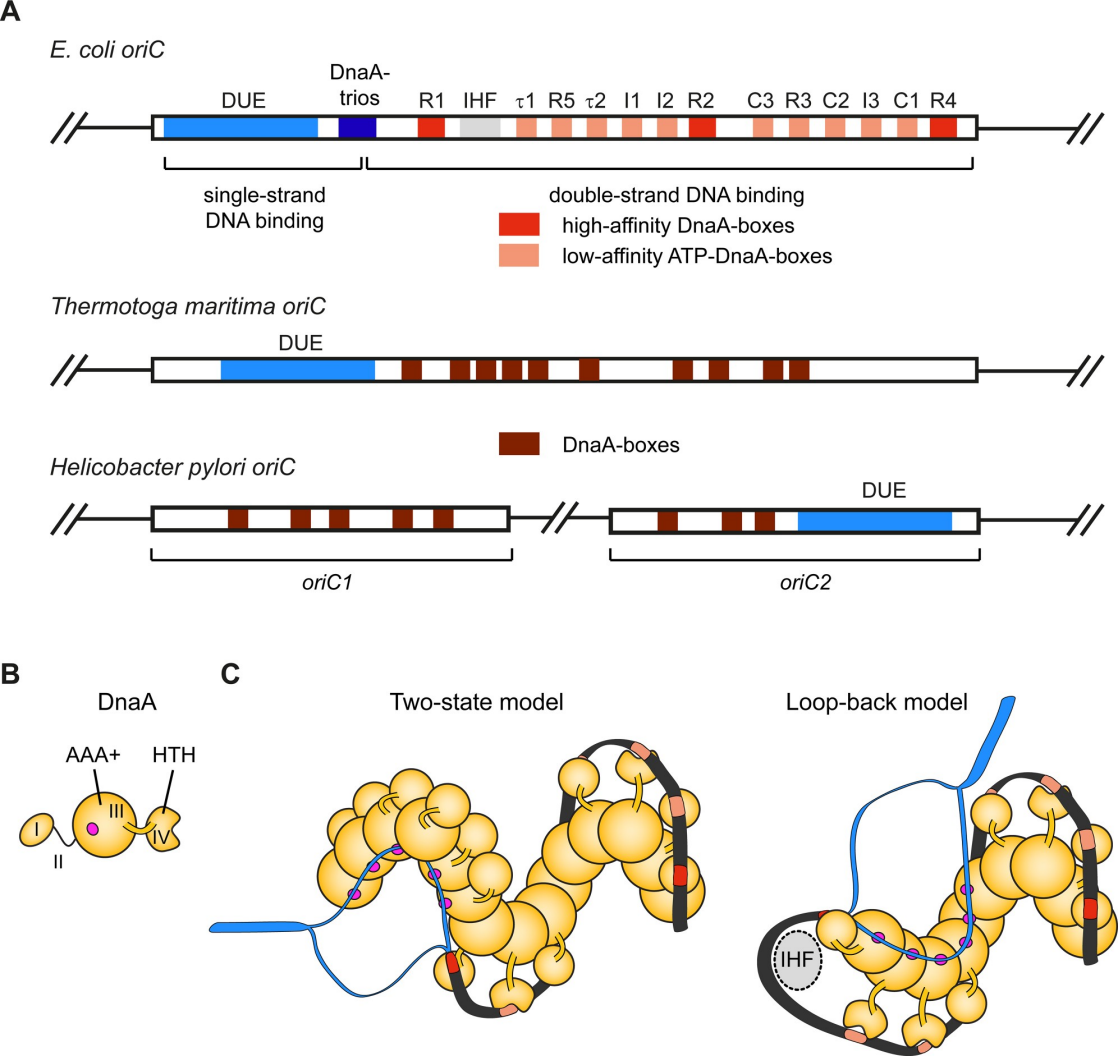
Origin organization and recognition in bacteria. **A**) Schematic of the architecture of *E*. *coli* origin *oriC*, *Thermotoga maritima oriC*, and the bipartite origin in *Helicobacter pylori*. The DUE is flanked on one side by several high- and weak-affinity DnaA-boxes as indicated for *E*. *coli oriC*. **B**) Domain organization of the *E*. *coli* initiator DnaA. The magenta circle indicates the single-strand DNA binding site. **C**) Models for origin recognition and melting by DnaA. In the two-state model (left panel), the DnaA protomers transition from a dsDNA binding mode (mediated by the HTH-domains recognizing DnaA-boxes) to an ssDNA binding mode (mediated by the AAA+ domains). In the loop-back model, the DNA is sharply bent backwards onto the DnaA filament (facilitated by the regulatory protein IHF [40]) so that a single protomer binds both duplex and single-stranded regions. In either instance, the DnaA filament melts the DNA duplex and stabilizes the initiation bubble prior to loading of the replicative helicase (DnaB in *E*. *coli*). HTH–helix-turn-helix domain, DUE–DNA unwinding element, IHF–integration host factor.

Most bacterial chromosomes are circular and contain a single origin of chromosomal replication (*oriC*). Bacterial *oriC* regions are surprisingly diverse in size (ranging from 250 bp to 2 kbp), sequence, and organization [41][42]; nonetheless, their ability to drive replication onset typically depends on sequence-specific readout of consensus DNA elements by the bacterial initiator, a protein called DnaA [43][44][45][46]. Origins in bacteria are either continuous or bipartite and contain three functional elements that control origin activity: conserved DNA repeats that are specifically recognized by DnaA (called DnaA-boxes), an AT-rich DNA unwinding element (DUE), and binding sites for proteins that help regulate replication initiation (for reviews, see [13][47][48]; **Fig 2A**). Interactions of DnaA both with the double-stranded (ds) DnaA-box regions and with single-stranded (ss) DNA in the DUE are important for origin activation and are mediated by different domains in the initiator protein: a helix-turn-helix (HTH) DNA binding element and an ATPase associated with various cellular activities (AAA+) domain, respectively (**Fig 2B**) [49][50][51][52][53][54][55][56]. While the sequence, number, and arrangement of origin-associated DnaA-boxes vary throughout the bacterial kingdom, their specific positioning and spacing in a given species are critical for *oriC* function and for productive initiation complex formation [41][42][57][58][59][60][61].

Among bacteria, *E*. *coli* is a particularly powerful model system to study the organization, recognition, and activation mechanism of replication origins. *E*. *coli oriC* comprises an approximately 260 bp region containing four types of initiator binding elements that differ in their affinities for DnaA and their dependencies on the co-factor ATP (**Fig 2A**). DnaA-boxes R1, R2, and R4 constitute high-affinity sites that are bound by the HTH domain of DnaA irrespective of the nucleotide-binding state of the initiator [43][62][63][64][65][66]. By contrast, the I, τ, and C-sites, which are interspersed between the R-sites, are low-affinity DnaA-boxes and associate preferentially with ATP-bound DnaA, although ADP-DnaA can substitute for ATP-DnaA under certain conditions [67][68][69][60]. Binding of the HTH domains to the high- and low-affinity DnaA recognition elements promotes ATP-dependent higher-order oligomerization of DnaA’s AAA+ modules into a right-handed filament that wraps duplex DNA around its outer surface, thereby generating superhelical torsion that facilitates melting of the adjacent AT-rich DUE (**Fig 2C**) [49][70][71][72]. DNA strand separation is additionally aided by direct interactions of DnaA’s AAA+ ATPase domain with triplet repeats, so-called DnaA-trios, in the proximal DUE region [73]. The engagement of single-stranded trinucleotide segments by the initiator filament stretches DNA and stabilizes the initiation bubble by preventing reannealing [53]. The DnaA-trio origin element is conserved in many bacterial species, indicating it is a key element for origin function [73]. After melting, the DUE provides an entry site for the *E*. *coli* replicative helicase DnaB, which is deposited onto each of the single DNA strands by its loader protein DnaC.

Although the different DNA binding activities of DnaA have been extensively studied biochemically and various *apo*, ssDNA-, or dsDNA-bound structures have been determined [52][53][54][71], the exact architecture of the higher-order DnaA-*oriC* initiation assembly remains unclear. Two models have been proposed to explain the organization of essential origin elements and DnaA-mediated *oriC* melting. The two-state model assumes a continuous DnaA filament that switches from a dsDNA binding mode (the organizing complex) to an ssDNA binding mode in the DUE (the melting complex) (**Fig 2C, left panel**) [71][74]. By contrast, in the loop-back model, the DNA is sharply bent in *oriC* and folds back onto the initiator filament so that DnaA protomers simultaneously engage double- and single-stranded DNA regions (**Fig 2C, right panel**) [75]. Elucidating how exactly *oriC* DNA is organized by DnaA remains thus an important task for future studies. Insights into initiation complex architecture will help explain not only how origin DNA is melted, but also how a replicative helicase is loaded directionally onto each of the exposed single DNA strands in the unwound DUE, and how these events are aided by interactions of the helicase with the initiator and specific loader proteins.

## Archaeal replication origins

**Fig 3:**
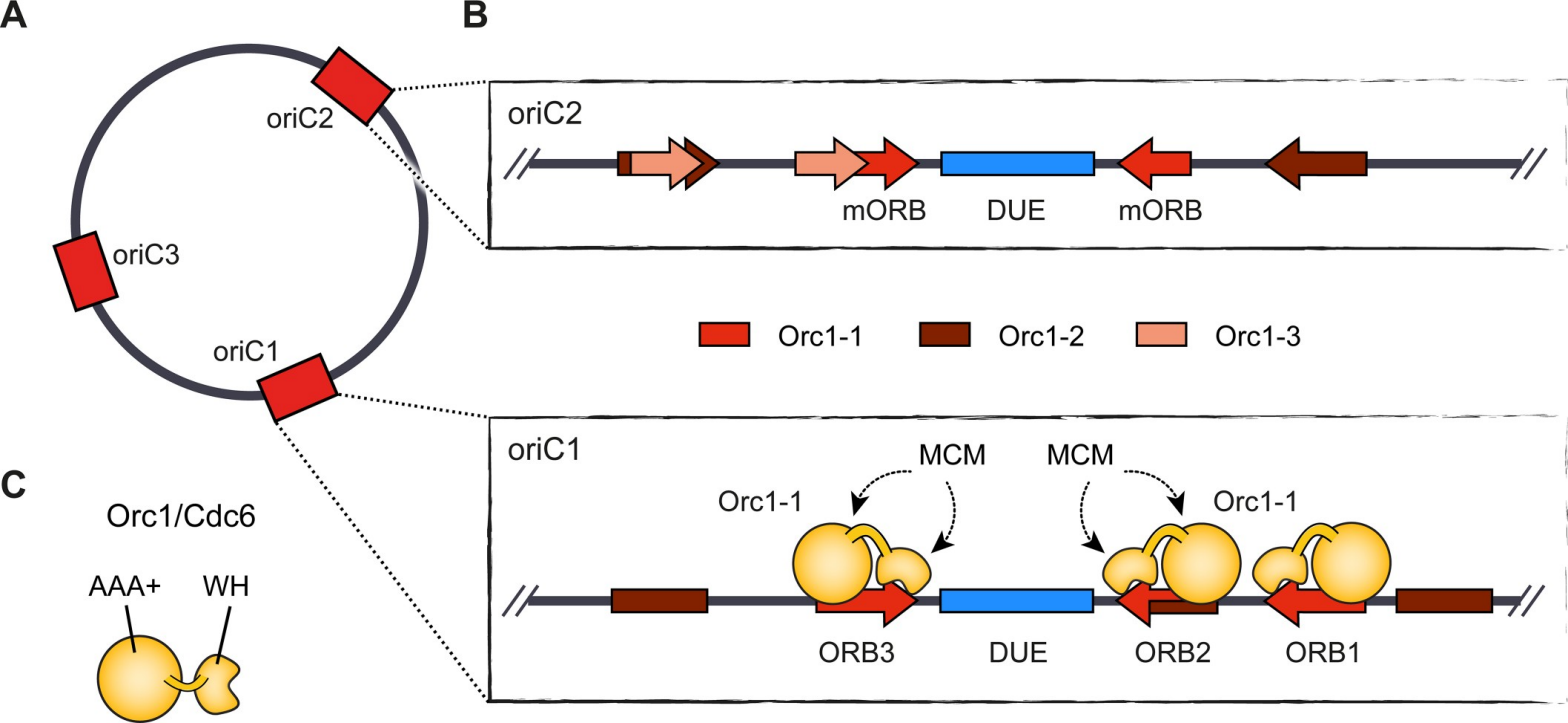
Origin organization and recognition in archaea. **A**) The circular chromosome of *Sulfolobus solfataricus* contains three different origins. **B**) Arrangement of initiator binding sites at two *S*. *solfataricus* origins, oriC1 and oriC2. Orc1-1 association with ORB elements is shown for oriC1. Recognition elements for additional Orc1/Cdc6 paralogs are also indicated, while WhiP binding sites have been omitted. **C**) Domain architecture of archaeal Orc1/Cdc6 paralogs. The orientation of ORB elements at origins leads to directional binding of Orc1/Cdc6 and MCM loading in between opposing ORBs (in **B**). (m)ORB–(mini-)origin recognition box, DUE–DNA unwinding element, WH–winged-helix domain.

Archaeal replication origins share some but not all of the organizational features of bacterial *oriC*. Unlike bacteria, archaea often initiate replication from multiple origins per chromosome (one to four have been reported) [76][77][78][79][80][81][82] [83][42]; yet, archaeal origins also bear specialized sequence regions that control origin function (for recent reviews, see [84][85][86]). These elements include both DNA sequence-specific origin recognition boxes (ORBs or miniORBs) and an AT-rich DUE that is flanked by one or several ORB regions [82][87]. ORB elements display a considerable degree of diversity in terms of their number, arrangement, and sequence, both among different archaeal species and among different origins within in a single species [77][82][88]. An additional degree of complexity is introduced by the initiator, Orc1/Cdc6 in archaea, which binds to ORB regions. Archaeal genomes typically encode multiple paralogs of Orc1/Cdc6 that vary substantially in their affinities for distinct ORB elements and that differentially contribute to origin activities [82][89][90][91]. In *Sulfolobus solfataricus*, for example, three chromosomal origins have been mapped (oriC1, oriC2, and oriC3; **Fig 3A**), and biochemical studies have revealed complex binding patterns of initiators at these sites (**Fig 3B**) [82][83][92][93]. The cognate initiator for oriC1 is Orc1-1, which associates with several ORBs at this origin [82][90]. OriC2 and oriC3 are bound by both Orc1-1 and Orc1-3 [82][90][93]. Conversely, a third paralog, Orc1-2, footprints at all three origins but has been postulated to negatively regulate replication initiation [82][93]. Additionally, the WhiP protein, an initiator unrelated to Orc1/Cdc6, has been shown to bind all origins as well and to drive origin activity of oriC3 in the closely related *Sulfolobus islandicus* [90][92]. Because archaeal origins often contain several adjacent ORB elements, multiple Orc1/Cdc6 paralogs can be simultaneously recruited to an origin and oligomerize in some instances [91][94]; however, in contrast to bacterial DnaA, formation of a higher-order initiator assembly does not appear to be a general prerequisite for origin function in the archaeal domain.

Structural studies have provided insights into how archaeal Orc1/Cdc6 recognizes ORB elements and remodels origin DNA [94][95]. Orc1/Cdc6 paralogs are two-domain proteins and are composed of a AAA+ ATPase module fused to a C-terminal winged-helix fold (**Fig 3C**) [96][97][98]. DNA-complexed structures of Orc1/Cdc6 revealed that ORBs are bound by an Orc1/Cdc6 monomer despite the presence of inverted repeat sequences within ORB elements [94][95]. Both the ATPase and winged-helix regions interact with the DNA duplex but contact the palindromic ORB repeat sequence asymmetrically, which orients Orc1/Cdc6 in a specific direction on the repeat [94][95]. Interestingly, the DUE-flanking ORB or miniORB elements often have opposite polarities [77][82][91][99][100], which predicts that the AAA+ lid subdomains and the winged-helix domains of Orc1/Cdc6 are positioned on either side of the DUE in a manner where they face each other (**Fig 3B, bottom panel**) [94][95]. Since both regions of Orc1/Cdc6 associate with the minichromosome maintenance (MCM) replicative helicase [101][102], this specific arrangement of ORB elements and Orc1/Cdc6 is likely important for loading two MCM complexes symmetrically onto the DUE (**Fig 3B**) [82]. Surprisingly, while the ORB DNA sequence determines the directionality of Orc1/Cdc6 binding, the initiator makes relatively few sequence-specific contacts with DNA [94][95]. However, Orc1/Cdc6 underwinds and bends DNA, suggesting that it relies on a mix of both DNA sequence and context-dependent DNA structural features to recognize origins [94][95][103]. Notably, base pairing is maintained in the distorted DNA duplex upon Orc1/Cdc6 binding in the crystal structures [94][95], whereas biochemical studies have yielded contradictory findings as to whether archaeal initiators can melt DNA similarly to bacterial DnaA [90][91][104]. Although the evolutionary kinship of archaeal and eukaryotic initiators and replicative helicases indicates that archaeal MCM is likely loaded onto duplex DNA (see next section), the temporal order of origin melting and helicase loading, as well as the mechanism for origin DNA melting, in archaeal systems remains therefore to be clearly established. Likewise, how exactly the MCM helicase is loaded onto DNA needs to be addressed in future studies.

## Eukaryotic replication origins

Origin organization, specification, and activation in eukaryotes are more complex than in bacterial or archaeal kingdoms and significantly deviate from the paradigm established for prokaryotic replication initiation. The large genome sizes of eukaryotic cells, which range from 12 Mbp in *S*. *cerevisiae* to 3 Gbp in humans, necessitates that DNA replication starts at several hundred (in budding yeast) to tens of thousands (in humans) origins to complete DNA replication of all chromosomes during each cell cycle (for recent reviews, see [32][23]). With the exception of *S*. *cerevisiae* and related *Saccharomycotina* species, eukaryotic origins do not contain consensus DNA sequence elements but their location is influenced by contextual cues such as local DNA topology, DNA structural features, and chromatin environment [25][31][33]. Nonetheless, eukaryotic origin function still relies on a conserved initiator protein complex to load replicative helicases onto DNA during the late M and G1 phases of the cell cycle, a step known as origin licensing (**Fig 1B**). [107] In contrast to their bacterial counterparts, replicative helicases in eukaryotes are loaded onto origin duplex DNA in an inactive, double-hexameric form and only a subset of them (10–20% in mammalian cells) is activated during any given S phase, events that are referred to as origin firing (**Fig 1B**) [108][109][110]. The location of active eukaryotic origins is therefore determined on at least two different levels, origin licensing to mark all potential origins, and origin firing to select a subset that permits assembly of the replication machinery and initiation of DNA synthesis. The extra licensed origins serve as backup and are activated only upon slowing or stalling of nearby replication forks, ensuring that DNA replication can be completed when cells encounter replication stress [111][112]. Together, the excess of licensed origins and the tight cell cycle control of origin licensing and firing embody two important strategies to prevent under- and overreplication and to maintain the integrity of eukaryotic genomes.

**Fig 4:**
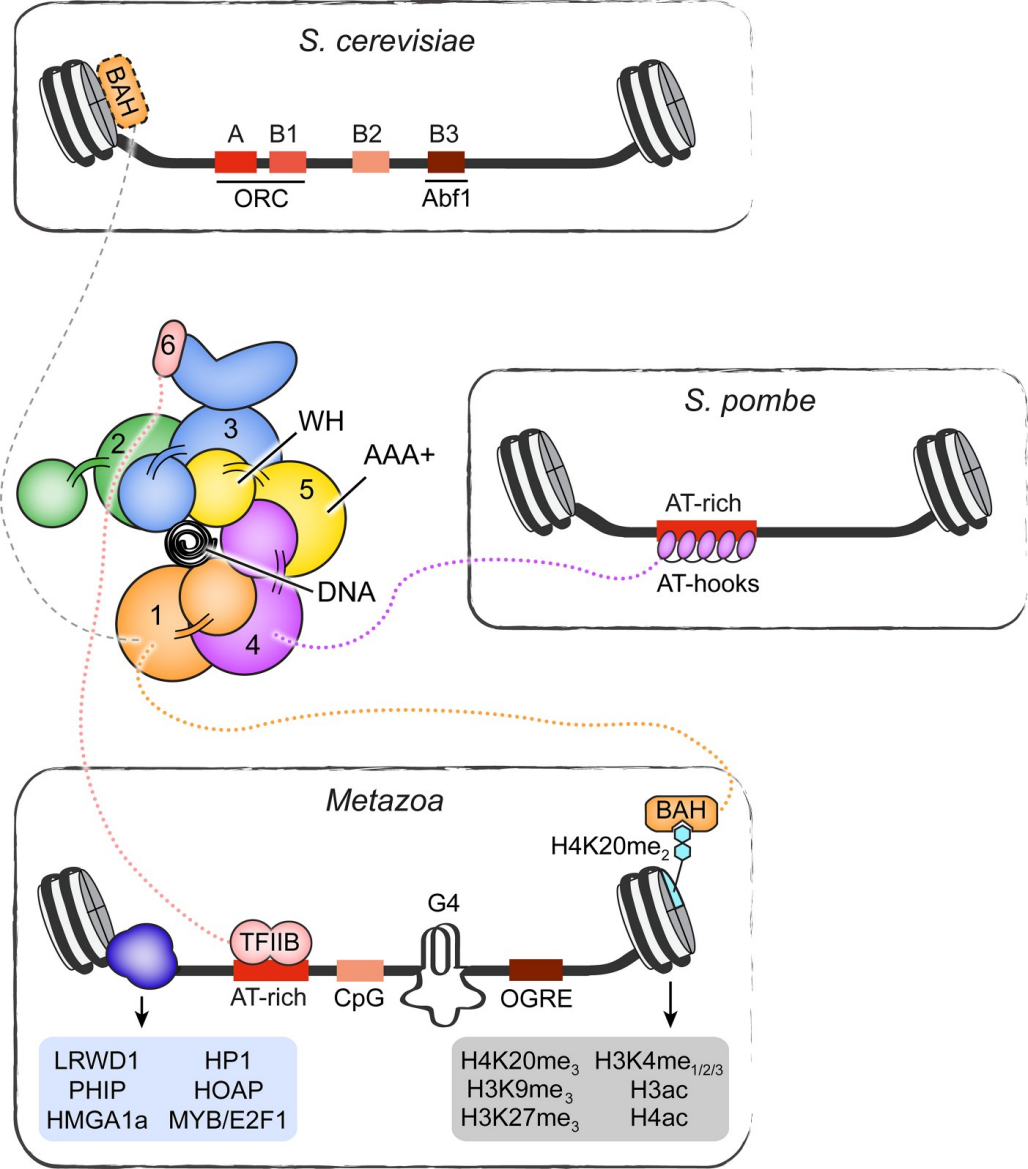
Origin organization and recognition in eukaryotes. Specific DNA elements and epigenetic features involved in ORC recruitment and origin function are summarized for *S*. *cerevisiae*, *S*. *pombe*, and metazoan origins. A schematic of the ORC architecture is also shown, highlighting the arrangement of the AAA+ and winged-helix domains into a pentameric ring that encircles origin DNA. Ancillary domains of several ORC subunits involved in targeting ORC to chromosomes are included. Other regions in ORC subunits may also be involved in initiator recruitment, either by directly or indirectly associating with partner proteins. A few examples are listed. Note that the BAH domain in *S*. *cerevisiae* Orc1 binds nucleosomes [105] but does not recognize H4K20me2 [106]. BAH–bromo-adjacent homology domain, WH–winged-helix domain, TFIIB–transcription factor II B-like domain in Orc6, G4 –G quadruplex, OGRE–origin G-rich repeated element.

Early studies in *S*. *cerevisiae* indicated that replication origins in eukaryotes might be recognized in a DNA-sequence-specific manner analogously to those in prokaryotes. In budding yeast, the search for genetic replicators lead to the identification of autonomously replicating sequences (ARS) that support efficient DNA replication initiation of extrachromosomal DNA [113][114][115]. These ARS regions are approximately 100–200 bp long and exhibit a multipartite organization, containing A, B1, B2, and sometimes B3 elements that together are essential for origin function (**Fig 4**) [116][117]. The A element encompasses the conserved 11 bp ARS consensus sequence (ACS) [118][119], which, in conjunction with the B1 element, constitutes the primary binding site for the heterohexameric origin recognition complex (ORC), the eukaryotic replication initiator [120][121][122][123]. Within ORC, five subunits are predicated on conserved AAA+ ATPase and winged-helix folds and co-assemble into a pentameric ring that encircles DNA (**Fig 4**) [123][124][125]. In budding yeast ORC, DNA binding elements in the ATPase and winged-helix domains, as well as adjacent basic patch regions in some of the ORC subunits, are positioned in the central pore of the ORC ring such that they aid the DNA-sequence-specific recognition of the ACS in an ATP-dependent manner [123][126]. By contrast, the roles of the B2 and B3 elements are less clear. The B2 region is similar to the ACS in sequence and has been suggested to function as a second ORC binding site under certain conditions, or as a binding site for the replicative helicase core [127][128][129][130][131]. Conversely, the B3 element recruits the transcription factor Abf1, albeit B3 is not found at all budding yeast origins and Abf1 binding does not appear to be strictly essential for origin function [116][132][133].

Origin recognition in eukaryotes other than *S*. *cerevisiae* or its close relatives does not conform to the sequence-specific readout of conserved origin DNA elements. Pursuits to isolate specific chromosomal replicator sequences more generally in eukaryotic species, either genetically or by genome-wide mapping of initiator binding or replication start sites, have failed to identify clear consensus sequences at origins [134][135][136][137][138][139][140][141][142][143][144][145]. Thus, sequence-specific DNA-initiator interactions in budding yeast signify a specialized mode for origin recognition in this system rather than an archetypal mode for origin specification across the eukaryotic domain. Nonetheless, DNA replication does initiate at discrete sites that are not randomly distributed across eukaryotic genomes, arguing that alternative means determine the chromosomal location of origins in these systems. These mechanisms involve a complex interplay between DNA accessibility, nucleotide sequence skew (both AT-richness and CpG islands have been linked to origins), nucleosome positioning, epigenetic features, DNA topology and certain DNA structural features (e.g., G4 motifs), as well as regulatory proteins and transcriptional interference [13][14][30][31][33][146][147][139][148]. Importantly, origin properties vary not only between different origins in an organism and among species, but some can also change during development and cell differentiation. The chorion locus in *Drosophila* follicle cells constitutes a well-established example for spatial and developmental control of initiation events. This region undergoes DNA-replication-dependent gene amplification at a defined stage during oogenesis and relies on the timely and specific activation of chorion origins, which in turn is regulated by origin-specific cis-elements and several protein factors, including the Myb complex, E2F1, and E2F2 [149][150][151][152][153]. This combinatorial specification and multifactorial regulation of metazoan origins has complicated the identification of unifying features that determine the location of replication start sites across eukaryotes more generally.

To facilitate replication initiation, ORC assemblies from various species have evolved specialized auxiliary domains that are thought to aid initiator targeting to chromosomal origins or chromosomes in general (**Fig 4**). For example, the Orc4 subunit in *S*. *pombe* ORC contains several AT-hooks that preferentially bind AT-rich DNA [154], while in metazoan ORC the TFIIB-like domain of Orc6 is thought to perform a similar function [155]. Metazoan Orc1 proteins also harbor a bromo-adjacent homology (BAH) domain that interacts with H4K20me2-nucleosomes [106]. Particularly in mammalian cells, H4K20 methylation has been reported to be required for efficient replication initiation, and the Orc1-BAH domain facilitates ORC association with chromosomes and Epstein-Barr virus origin-dependent replication [156][157][158][159][160]. Therefore, it is intriguing to speculate that both observations are mechanistically linked at least in a subset of metazoa, but this possibility needs to be further explored in future studies. In addition to the recognition of certain DNA or epigenetic features, ORC also associates directly or indirectly with several partner proteins that could aid initiator recruitment, including LRWD1, PHIP (or DCAF14), HMGA1a, among others (**Fig 4**) [29][161][162][163][164][165][166][167]. Interestingly, *Drosophila* ORC, like its budding yeast counterpart, bends DNA and negative supercoiling has been reported to enhance DNA binding of this complex, suggesting that DNA topology and malleability might influence the location of ORC binding sites across metazoan genomes [27][123][168][169][170]. A molecular understanding for how ORC’s DNA binding regions might support the readout of structural properties of the DNA duplex in metazoans rather than of specific DNA sequences as in *S*. *cerevisiae* awaits high-resolution structural information of DNA-bound metazoan initiator assemblies. Likewise, how different epigenetic factors contribute to initiator recruitment in metazoan systems is poorly defined and is an important question that needs to be addressed in more detail.

Once recruited to origins, ORC and its co-factors Cdc6 and Cdt1 drive the deposition of the minichromosome maintenance 2–7 (Mcm2-7) complex onto DNA (for reviews see [107][171]). Like the archaeal replicative helicase core, Mcm2-7 is loaded as a head-to-head double hexamer onto DNA to license origins (**Fig 1B**) [108][109][110]. In S-phase, Dbf4-dependent kinase (DDK) and cyclin-dependent kinase (CDK) phosphorylate several Mcm2-7 subunits and additional initiation factors to promote the recruitment of the helicase co-activators Cdc45 and GINS, DNA melting, and ultimately bidirectional replisome assembly at a subset of the licensed origins (**Fig 1B**) [172][24]. In both yeast and metazoans, origins are free or depleted of nucleosomes, a property that is crucial for Mcm2-7 loading, indicating that chromatin state at origins regulates not only initiator recruitment but also helicase loading [140][173][174][175][176][177]. A permissive chromatin environment is further important for origin activation and has been implicated in regulating both origin efficiency and the timing of origin firing. Euchromatic origins typically contain active chromatin marks, replicate early, and are more efficient than late-replicating, heterochromatic origins, which conversely are characterized by repressive marks [23][175][178]. Not surprisingly, several chromatin remodelers and chromatin-modifying enzymes have been found to associate with origins and certain initiation factors [179][180], but how their activities impact different replication initiation events remains largely obscure. Remarkably, cis-acting “early replication control elements” (ERCEs) have recently also been identified to help regulate replication timing and to influence 3D genome architecture in mammalian cells [181]. Understanding the molecular and biochemical mechanisms that orchestrate this complex interplay between 3D genome organization, local and higher-order chromatin structure, and replication initiation is an exciting topic for further studies.

Why have metazoan replication origins diverged from the DNA sequence-specific recognition paradigm that determines replication start sites in prokaryotes and budding yeast? Observations that metazoan origins often co-localize with promoter regions in *Drosophila* and mammalian cells and that replication-transcription conflicts due to collisions of the underlying molecular machineries can lead to DNA damage suggest that proper coordination of transcription and replication is important for maintaining genome stability [135][137][139][142][182][16][17][19]. Recent findings also point to a more direct role of transcription in influencing the location of origins, either by inhibiting Mcm2-7 loading or by repositioning of loaded Mcm2-7 on chromosomes [183][148]. Sequence-independent (but not necessarily random) initiator binding to DNA additionally allows for flexibility in specifying helicase loading sites and, together with transcriptional interference and the variability in activation efficiencies of licensed origins, likely determines origin location and contributes to the co-regulation of DNA replication and transcriptional programs during development and cell fate transitions. Computational modeling of initiation events in *S*. *pombe*, as well as the identification of cell-type specific and developmentally-regulated origins in metazoans, are in agreement with this notion [136][144][184][185][186][187][188][148]. However, a large degree of flexibility in origin choice also exists among different cells within a single population [139][145][185], and the molecular mechanisms that lead to the heterogeneity in origin usage remain ill-defined. Mapping origins in single cells in metazoan systems and correlating these initiation events with single-cell gene expression and chromatin status will be important to elucidate whether origin choice is purely stochastic or controlled in a defined manner.

## Concluding remarks

Although DNA replication is essential for genetic inheritance, defined, site-specific replication origins are technically not a requirement for genome duplication as long as all chromosomes are copied in their entirety to maintain gene copy numbers. Certain bacteriophages and viruses, for example, can initiate DNA replication by homologous recombination independent of dedicated origins [189]. Likewise, the archaeon *Haloferax volcanii* uses recombination-dependent initiation to duplicate its genome when its endogenous origins are deleted [78]. Similar non-canonical initiation events through break-induced or transcription-initiated replication have been reported in *E*. *coli* and *S*. *cerevisiae* [190][191][192][193][194]. Nonetheless, despite the ability of cells to sustain viability under these exceptional circumstances, origin-dependent initiation is a common strategy universally adopted across different domains of life. The controlled assembly of the replication machinery at origins likely confers long-term advantage to cells by allowing tight cell cycle regulation and by maintaining a specific replication dynamics. The divergent origin specification modes between prokaryotes and budding yeast on the one hand and metazoans on the other hand appear to reflect distinct needs to coordinate the spatiotemporal replication program with gene expression and cell differentiation programs to ensure not only genetic but also epigenetic inheritance and to preserve cell identity. Deciphering the underlying molecular mechanisms that modulate origin location, usage, and timing to define the replication program in metazoan systems represents an important major challenge in the field and will be essential to understand how dysregulation of these events are linked to human diseases. In addition, detailed studies of replication initiation have focused on a limited number of model systems. The extensively studied fungi and metazoa are both members of the opisthokont supergroup and exemplify only a small fraction of the evolutionary landscape in the eukaryotic domain [195]. Comparably few efforts have been directed at other eukaryotic model systems, such as kinetoplastids or tetrahymena [196][197][198][199][200] [201][202]. Surprisingly, these studies have revealed interesting differences both in origin properties and in initiator composition compared to yeast and metazoans. Further exploration of replication initiation mechanisms across different branches of the eukaryotic domain will likely yield unexpected insight into the diversity and evolution of this fundamental biological process.

## Supporting information

S1 Text Version history of the text file. (XML)

S2 Text Peer reviews and response to reviews. (XML)
