# Correction: Origins of DNA replication

**DOI:** 10.1371/journal.pgen.1008556

**Published:** 2019-12-19

**Authors:** The PLOS Genetics Staff

There are numerous errors in the Reference section, which were introduced by the PLOS production team. Specifically, there are errors in the formatting and spacing of the journal names contained within the references. The publisher apologizes for the errors. The corrected reference list can be found here:

1. Mendel G (1865) Versuche ueber Pflanzenhybriden *Verhandlungen des naturforschenden Vereines in Bruenn*, *Bd IV* Abhandlungen:3–47

2. Avery OT, Macleod CM & McCarty M (1944) Studies on the chemical nature of the substance inducing transformation of pneumococcal types: induction of transformation by a deoxyribonucleic acid fraction isolated from pneumococcus type iii *J*. *Exp*. *Med*. 79:137–58 [PMID: 19871359]

3. Watson JD & Crick FH (1953) The structure of DNA *Cold Spring Harb*. *Symp*. *Quant*. *Biol*. 18:123–31 [PMID: 13168976]

4. Meselson M & Stahl FW (1958) The replication of DNA in Escherichia coli *Proc*. *Natl*. *Acad*. *Sci*. *U*.*S*.*A*. 44:671–82 [PMID: 16590258]

5. Meselson M & Stahl FW (1958) The replication of DNA *Cold Spring Harb*. *Symp*. *Quant*. *Biol*. 23:9–12 [PMID: 13635537]

6. Lehman IR, Bessman MJ, Simms ES & Kornberg A (1958) Enzymatic synthesis of deoxyribonucleic acid. I. Preparation of substrates and partial purification of an enzyme from Escherichia coli *J*. *Biol*. *Chem*. 233:163–70 [PMID: 13563462]

7. O'Donnell M, Langston L & Stillman B (2013) Principles and concepts of DNA replication in bacteria, archaea, and eukarya *Cold Spring Harb*. *Perspect*. *Biol*. 5:a010108 [PMID: 23818497][DOI]

8. Barlow JH & Nussenzweig A (2014) Replication initiation and genome instability: a crossroads for DNA and RNA synthesis *Cell*. *Mol*. *Life Sci*. 71:4545–59 [PMID: 25238783][DOI]

9. Abbas T, Keaton MA & Dutta A (2013) Genomic instability in cancer *Cold Spring Harb*. *Perspect*. *Biol*. 5:a012914 [PMID: 23335075][DOI]

10. Siddiqui K, On KF & Diffley JF (2013) Regulating DNA replication in eukarya *Cold Spring Harb*. *Perspect*. *Biol*. 5:a012930 [PMID: 23838438][DOI]

11. Sclafani RA & Holzen TM (2007) Cell cycle regulation of DNA replication *Annu*. *Rev*. *Genet*. 41:237–80 [PMID: 17630848][DOI]

12. García-Muse T & Aguilera A (2016) Transcription-replication conflicts: how they occur and how they are resolved *Nat*. *Rev*. *Mol*. *Cell Biol*. 17:553–63 [PMID: 27435505][DOI]

13. Leonard AC & Méchali M (2013) DNA replication origins *Cold Spring Harb*. *Perspect*. *Biol*. 5:a010116 [PMID: 23838439][DOI]

14. Creager RL, Li Y & MacAlpine DM (2015) SnapShot: Origins of DNA replication *Cell* 161:418-418.e1 [PMID: 25860614][DOI]

15. Knott SR, Viggiani CJ & Aparicio OM (2009) To promote and protect: coordinating DNA replication and transcription for genome stability *Epigenetics* 4:362–5 [PMID: 19736523]

16. Deshpande AM & Newlon CS (1996) DNA replication fork pause sites dependent on transcription *Science* 272:1030–3 [PMID: 8638128]

17. Sankar TS, Wastuwidyaningtyas BD, Dong Y, Lewis SA & Wang JD (2016) The nature of mutations induced by replication–transcription collisions *Nature* 535:178–81 [PMID: 27362223][DOI]

18. Liu B & Alberts BM (1995) Head-on collision between a DNA replication apparatus and RNA polymerase transcription complex *Science* 267:1131–7 [PMID: 7855590]

19. Azvolinsky A, Giresi PG, Lieb JD & Zakian VA (2009) Highly transcribed RNA polymerase II genes are impediments to replication fork progression in Saccharomyces cerevisiae *Mol*. *Cell* 34:722–34 [PMID: 19560424][DOI]

20. Jacob F, Brenner S & Cuzin F (1963) On the regulation of DNA replication in bacteria *Cold Spring Harbor Symp*. *Quant*. *Biol*. 28:329–48

21. Novick RP (1987) Plasmid incompatibility *Microbiol*. *Rev*. 51:381–95 [PMID: 3325793]

22. Skarstad K & Katayama T (2013) Regulating DNA replication in bacteria *Cold Spring Harb*. *Perspect*. *Biol*. 5:a012922 [PMID: 23471435][DOI]

23. Marks AB, Fu H & Aladjem MI (2017) Regulation of Replication Origins *Adv*. *Exp*. *Med*. *Biol*. 1042:43–59 [PMID: 29357052][DOI]

24. Parker MW, Botchan MR & Berger JM (2017) Mechanisms and regulation of DNA replication initiation in eukaryotes *Crit*. *Rev*. *Biochem*. *Mol*. *Biol*. 52:107–44 [PMID: 28094588][DOI]

25. Gilbert DM (2004) In search of the holy replicator *Nat*. *Rev*. *Mol*. *Cell Biol*. 5:848–55 [PMID: 15459665][DOI]

26. Aladjem MI & Fanning E (2004) The replicon revisited: an old model learns new tricks in metazoan chromosomes *EMBO Rep*. 5:686–91 [PMID: 15229645][DOI]

27. Remus D, Beall EL & Botchan MR (2004) DNA topology, not DNA sequence, is a critical determinant for Drosophila ORC-DNA binding *EMBO J*. 23:897–907 [PMID: 14765124][DOI]

28. Vashee S, Cvetic C, Lu W, Simancek P, Kelly TJ & Walter JC (2003) Sequence-independent DNA binding and replication initiation by the human origin recognition complex *Genes Dev*. 17:1894–908 [PMID: 12897055][DOI]

29. Shen Z, Sathyan KM, Geng Y, Zheng R, Chakraborty A, Freeman B, Wang F, Prasanth KV & Prasanth SG (2010) A WD-repeat protein stabilizes ORC binding to chromatin *Mol*. *Cell* 40:99–111 [PMID: 20932478][DOI]

30. Dorn ES & Cook JG (2011) Nucleosomes in the neighborhood: new roles for chromatin modifications in replication origin control *Epigenetics* 6:552–9 [PMID: 21364325]

31. Aladjem MI & Redon CE (2017) Order from clutter: selective interactions at mammalian replication origins *Nat*. *Rev*. *Genet*. 18:101–16 [PMID: 27867195][DOI]

32. Fragkos M, Ganier O, Coulombe P & Méchali M (2015) DNA replication origin activation in space and time *Nat*. *Rev*. *Mol*. *Cell Biol*.16:360–74 [PMID: 25999062][DOI]

33. Prioleau MN & MacAlpine DM (2016) DNA replication origins-where do we begin? *Genes Dev*. 30:1683–97 [PMID: 27542827][DOI]

34. Cayrou C, Coulombe P, Puy A, Rialle S, Kaplan N, Segal E & Méchali M (2012) New insights into replication origin characteristics in metazoans *Cell Cycle* 11:658–67 [PMID: 22373526][DOI]

35. Lombraña R, Almeida R, Álvarez A & Gómez M (2015) R-loops and initiation of DNA replication in human cells: a missing link? *Front*. *Genet*. 6:158 [PMID: 25972891][DOI]

36. Jang SM, Zhang Y, Utani K, Fu H, Redon CE, Marks AB, Smith OK, Redmond CJ, Baris AM, Tulchinsky DA & Aladjem MI (2018) The replication initiation determinant protein (RepID) modulates replication by recruiting CUL4 to chromatin *Nat*. *Commun*. 9:2782 [PMID: 30018425][DOI]

37. Zakian VA & Scott JF (1982) Construction, replication, and chromatin structure of TRP1 RI circle, a multiple-copy synthetic plasmid derived from Saccharomyces cerevisiae chromosomal DNA *Mol*. *Cell*. *Biol*. 2:221–32 [PMID: 6287231][DOI]

38. Rhodes N, Company M & Errede B (1990) A yeast-Escherichia coli shuttle vector containing the M13 origin of replication *Plasmid* 23:159–62 [PMID: 2194231]

39. Paululat A & Heinisch JJ (2012) New yeast/E. coli/Drosophila triple shuttle vectors for efficient generation of Drosophila P element transformation constructs *Gene* 511:300–5 [PMID: 23026211][DOI]

40. Ryan VT, Grimwade JE, Camara JE, Crooke E & Leonard AC (2004) Escherichia coli prereplication complex assembly is regulated by dynamic interplay among Fis, IHF and DnaA *Mol*. *Microbiol*. 51:1347–59 [PMID: 14982629][DOI]

41. Mackiewicz P, Zakrzewska-Czerwinska J, Zawilak A, Dudek MR & Cebrat S (2004) Where does bacterial replication start? Rules for predicting the oriC region *Nucleic Acids Res*. 32:3781–91 [PMID: 15258248][DOI]

42. Luo H & Gao F (2019) DoriC 10.0: an updated database of replication origins in prokaryotic genomes including chromosomes and plasmids *Nucleic Acids Res*. 47:D74-D77 [PMID: 30364951][DOI]

43. Fuller RS, Funnell BE & Kornberg A (1984) The dnaA protein complex with the E. coli chromosomal replication origin (oriC) and other DNA sites *Cell* 38:889–900 [PMID: 6091903]

44. Fuller RS & Kornberg A (1983) Purified dnaA protein in initiation of replication at the Escherichia coli chromosomal origin of replication *Proc*. *Natl*. *Acad*. *Sci*. *U*.*S*.*A*. 80:5817–21 [PMID: 6310593]

45. Jakimowicz D, Majka J, Messer W, Speck C, Fernandez M, Martin MC, Sanchez J, Schauwecker F, Keller U, Schrempf H & Zakrzewska-Czerwińska J (1998) Structural elements of the Streptomyces oriC region and their interactions with the DnaA protein *Microbiology* 144:1281–90 [PMID: 9611803][DOI]

46. Tsodikov OV & Biswas T (2011) Structural and thermodynamic signatures of DNA recognition by Mycobacterium tuberculosis DnaA *J*. *Mol*. *Biol*. 410:461–76 [PMID: 21620858][DOI]

47. Costa A, Hood IV & Berger JM (2013) Mechanisms for initiating cellular DNA replication *Annu*. *Rev*. *Biochem*. 82:25–54 [PMID: 23746253][DOI]

48. Wolański M, Donczew R, Zawilak-Pawlik A & Zakrzewska-Czerwińska J (2014) oriC-encoded instructions for the initiation of bacterial chromosome replication *Front*. *Microbiol*. 5:735 [PMID: 25610430][DOI]

49. Messer W, Blaesing F, Majka J, Nardmann J, Schaper S, Schmidt A, Seitz H, Speck C, Tüngler D, Wegrzyn G, Weigel C, Welzeck M & Zakrzewska-Czerwinska J (1999) Functional domains of DnaA proteins *Biochimie* 81:819–25 [PMID: 10572294]

50. Sutton MD & Kaguni JM (1997) The Escherichia coli dnaA gene: four functional domains *J*. *Mol*. *Biol*. 274:546–61 [PMID: 9417934][DOI]

51. Speck C & Messer W (2001) Mechanism of origin unwinding: sequential binding of DnaA to double- and single-stranded DNA *EMBO J*. 20:1469–76 [PMID: 11250912][DOI]

52. Fujikawa N, Kurumizaka H, Nureki O, Terada T, Shirouzu M, Katayama T & Yokoyama S (2003) Structural basis of replication origin recognition by the DnaA protein *Nucleic Acids Res*. 31:2077–86 [PMID: 12682358]

53. Duderstadt KE, Chuang K & Berger JM (2011) DNA stretching by bacterial initiators promotes replication origin opening *Nature* 478:209–13 [PMID: 21964332][DOI]

54. Erzberger JP, Pirruccello MM & Berger JM (2002) The structure of bacterial DnaA: implications for general mechanisms underlying DNA replication initiation *EMBO J*. 21:4763–73 [PMID: 12234917]

55. Iyer LM, Leipe DD, Koonin EV & Aravind L (2004) Evolutionary history and higher order classification of AAA+ ATPases *J*. *Struct*. *Biol*.146:11–31 [PMID: 15037234][DOI]

56. Sutton MD & Kaguni JM (1997) Threonine 435 of Escherichia coli DnaA protein confers sequence-specific DNA binding activity *J*. *Biol*. *Chem*. 272:23017–24 [PMID: 9287298]

57. Bramhill D & Kornberg A (1988) A model for initiation at origins of DNA replication *Cell* 54:915–8 [PMID: 2843291]

58. Rozgaja TA, Grimwade JE, Iqbal M, Czerwonka C, Vora M & Leonard AC (2011) Two oppositely oriented arrays of low-affinity recognition sites in oriC guide progressive binding of DnaA during Escherichia coli pre-RC assembly *Mol*. *Microbiol*. 82:475–88 [PMID: 21895796][DOI]

59. Zawilak-Pawlik A, Kois A, Majka J, Jakimowicz D, Smulczyk-Krawczyszyn A, Messer W & Zakrzewska-Czerwińska J (2005) Architecture of bacterial replication initiation complexes: orisomes from four unrelated bacteria *Biochem*. *J*. 389:471–81 [PMID: 15790315][DOI]

60. Grimwade JE, Rozgaja TA, Gupta R, Dyson K, Rao P & Leonard AC (2018) Origin recognition is the predominant role for DnaA-ATP in initiation of chromosome replication *Nucleic Acids Res*. 46:6140–51 [PMID: 29800247][DOI]

61. Sakiyama Y, Kasho K, Noguchi Y, Kawakami H & Katayama T (2017) Regulatory dynamics in the ternary DnaA complex for initiation of chromosomal replication in Escherichia coli *Nucleic Acids Res*. 45:12354–73 [PMID: 29040689][DOI]

62. Matsui M, Oka A, Takanami M, Yasuda S & Hirota Y (1985) Sites of dnaA protein-binding in the replication origin of the Escherichia coli K-12 chromosome *J*. *Mol*. *Biol*. 184:529–33 [PMID: 2995681]

63. Margulies C & Kaguni JM (1996) Ordered and sequential binding of DnaA protein to oriC, the chromosomal origin of Escherichia coli *J*. *Biol*. *Chem*. 271:17035–40 [PMID: 8663334]

64. Schaper S & Messer W (1995) Interaction of the initiator protein DnaA of Escherichia coli with its DNA target *J*. *Biol*. *Chem*. 270:17622–6 [PMID: 7615570]

65. Weigel C, Schmidt A, Rückert B, Lurz R & Messer W (1997) DnaA protein binding to individual DnaA boxes in the Escherichia coli replication origin, oriC *EMBO J*. 16:6574–83 [PMID: 9351837][DOI]

66. Samitt CE, Hansen FG, Miller JF & Schaechter M (1989) In vivo studies of DnaA binding to the origin of replication of Escherichia coli *EMBO J*. 8:989–93 [PMID: 2542031]

67. McGarry KC, Ryan VT, Grimwade JE & Leonard AC (2004) Two discriminatory binding sites in the Escherichia coli replication origin are required for DNA strand opening by initiator DnaA-ATP *Proc*. *Natl*. *Acad*. *Sci*. *U*.*S*.*A*. 101:2811–6 [PMID: 14978287][DOI]

68. Kawakami H, Keyamura K & Katayama T (2005) Formation of an ATP-DnaA-specific initiation complex requires DnaA Arginine 285, a conserved motif in the AAA+ protein family *J*. *Biol*. *Chem*. 280:27420–30 [PMID: 15901724][DOI]

69. Speck C, Weigel C & Messer W (1999) ATP- and ADP-dnaA protein, a molecular switch in gene regulation *EMBO J*. 18:6169–76 [PMID: 10545126][DOI]

70. Miller DT, Grimwade JE, Betteridge T, Rozgaja T, Torgue JJ & Leonard AC (2009) Bacterial origin recognition complexes direct assembly of higher-order DnaA oligomeric structures *Proc*. *Natl*. *Acad*. *Sci*. *U*.*S*.*A*. 106:18479–84 [PMID: 19833870][DOI]

71. Erzberger JP, Mott ML & Berger JM (2006) Structural basis for ATP-dependent DnaA assembly and replication-origin remodeling *Nat*. *Struct*. *Mol*. *Biol*. 13:676–83 [PMID: 16829961][DOI]

72. Zorman S, Seitz H, Sclavi B & Strick TR (2012) Topological characterization of the DnaA-oriC complex using single-molecule nanomanipuation *Nucleic Acids Res*. 40:7375–83 [PMID: 22581769][DOI]

73. Richardson TT, Harran O & Murray H (2016) The bacterial DnaA-trio replication origin element specifies single-stranded DNA initiator binding *Nature* 534:412–6 [PMID: 27281207][DOI]

74. Duderstadt KE, Mott ML, Crisona NJ, Chuang K, Yang H & Berger JM (2010) Origin remodeling and opening in bacteria rely on distinct assembly states of the DnaA initiator *J*. *Biol*. *Chem*. 285:28229–39 [PMID: 20595381][DOI]

75. Ozaki S & Katayama T (2012) Highly organized DnaA-oriC complexes recruit the single-stranded DNA for replication initiation *Nucleic Acids Res*. 40:1648–65 [PMID: 22053082][DOI]

76. Myllykallio H, Lopez P, López-García P, Heilig R, Saurin W, Zivanovic Y, Philippe H & Forterre P (2000) Bacterial mode of replication with eukaryotic-like machinery in a hyperthermophilic archaeon *Science* 288:2212–5 [PMID: 10864870]

77. Norais C, Hawkins M, Hartman AL, Eisen JA, Myllykallio H & Allers T (2007) Genetic and physical mapping of DNA replication origins in Haloferax volcanii *PLoS Genet*. 3:e77 [PMID: 17511521][DOI]

78. Hawkins M, Malla S, Blythe MJ, Nieduszynski CA & Allers T (2013) Accelerated growth in the absence of DNA replication origins *Nature* 503:544–7 [PMID: 24185008][DOI]

79. Wu Z, Liu J, Yang H, Liu H & Xiang H (2014) Multiple replication origins with diverse control mechanisms in Haloarcula hispanica *Nucleic Acids Res*. 42:2282–94 [PMID: 24271389][DOI]

80. Pelve EA, Martens-Habbena W, Stahl DA & Bernander R (2013) Mapping of active replication origins in vivo in thaum- and euryarchaeal replicons *Mol*. *Microbiol*. 90:538–50 [PMID: 23991938][DOI]

81. Pelve EA, Lindås AC, Knöppel A, Mira A & Bernander R (2012) Four chromosome replication origins in the archaeon Pyrobaculum calidifontis *Mol*. *Microbiol*. 85:986–95 [PMID: 22812406][DOI]

82. Robinson NP, Dionne I, Lundgren M, Marsh VL, Bernander R & Bell SD (2004) Identification of two origins of replication in the single chromosome of the archaeon Sulfolobus solfataricus *Cell* 116:25–38 [PMID: 14718164]

83. Lundgren M, Andersson A, Chen L, Nilsson P & Bernander R (2004) Three replication origins in Sulfolobus species: synchronous initiation of chromosome replication and asynchronous termination *Proc*. *Natl*. *Acad*. *Sci*. *U*.*S*.*A*. 101:7046–51 [PMID: 15107501][DOI]

84. Bell SD (2017) Initiation of DNA Replication in the Archaea *Adv*. *Exp*. *Med*. *Biol*. 1042:99–115 [PMID: 29357055][DOI]

85. Ausiannikava D & Allers T (2017) Diversity of DNA Replication in the Archaea *Genes (Basel)* 8:E56 [PMID: 28146124][DOI]

86. Wu Z, Liu J, Yang H & Xiang H (2014) DNA replication origins in archaea *Front*. *Microbiol*. 5:179 [PMID: 24808892][DOI]

87. Matsunaga F, Forterre P, Ishino Y & Myllykallio H (2001) In vivo interactions of archaeal Cdc6/Orc1 and minichromosome maintenance proteins with the replication origin *Proc*. *Natl*. *Acad*. *Sci*. *U*.*S*.*A*. 98:11152–7 [PMID: 11562464][DOI]

88. Wu Z, Liu H, Liu J, Liu X & Xiang H (2012) Diversity and evolution of multiple orc/cdc6-adjacent replication origins in haloarchaea *BMC Genomics* 13:478 [PMID: 22978470][DOI]

89. Bell SD (2012) Archaeal orc1/cdc6 proteins *Subcell*. *Biochem*. 62:59–69 [PMID: 22918580][DOI]

90. Samson RY, Xu Y, Gadelha C, Stone TA, Faqiri JN, Li D, Qin N, Pu F, Liang YX, She Q & Bell SD (2013) Specificity and function of archaeal DNA replication initiator proteins *Cell Rep*. 3:485–96 [PMID: 23375370][DOI]

91. Grainge I, Gaudier M, Schuwirth BS, Westcott SL, Sandall J, Atanassova N & Wigley DB (2006) Biochemical analysis of a DNA replication origin in the archaeon Aeropyrum pernix *J*. *Mol*. *Biol*. 363:355–69 [PMID: 16978641][DOI]

92. Robinson NP & Bell SD (2007) Extrachromosomal element capture and the evolution of multiple replication origins in archaeal chromosomes *Proc*. *Natl*. *Acad*. *Sci*. *U*.*S*.*A*. 104:5806–11 [PMID: 17392430][DOI]

93. Robinson NP, Blood KA, McCallum SA, Edwards PA & Bell SD (2007) Sister chromatid junctions in the hyperthermophilic archaeon Sulfolobus solfataricus *EMBO J*. 26:816–24 [PMID: 17255945][DOI]

94. Dueber EL, Corn JE, Bell SD & Berger JM (2007) Replication origin recognition and deformation by a heterodimeric archaeal Orc1 complex *Science* 317:1210–3 [PMID: 17761879][DOI]

95. Gaudier M, Schuwirth BS, Westcott SL & Wigley DB (2007) Structural basis of DNA replication origin recognition by an ORC protein *Science* 317:1213–6 [PMID: 17761880][DOI]

96. Capaldi SA & Berger JM (2004) Biochemical characterization of Cdc6/Orc1 binding to the replication origin of the euryarchaeon Methanothermobacter thermoautotrophicus *Nucleic Acids Res*. 32:4821–32 [PMID: 15358831][DOI]

97. Liu J, Smith CL, DeRyckere D, DeAngelis K, Martin GS & Berger JM (2000) Structure and function of Cdc6/Cdc18: implications for origin recognition and checkpoint control *Mol*. *Cell* 6:637–48 [PMID: 11030343]

98. Singleton MR, Morales R, Grainge I, Cook N, Isupov MN & Wigley DB (2004) Conformational changes induced by nucleotide binding in Cdc6/ORC from Aeropyrum pernix *J*. *Mol*. *Biol*. 343:547–57 [PMID: 15465044][DOI]

99. Matsunaga F, Norais C, Forterre P & Myllykallio H (2003) Identification of short 'eukaryotic' Okazaki fragments synthesized from a prokaryotic replication origin *EMBO Rep*. 4:154–8 [PMID: 12612604][DOI]

100. Berquist BR & DasSarma S (2003) An archaeal chromosomal autonomously replicating sequence element from an extreme halophile, Halobacterium sp. strain NRC-1 *J*. *Bacteriol*. 185:5959–66 [PMID: 14526006]

101. Kasiviswanathan R, Shin JH & Kelman Z (2005) Interactions between the archaeal Cdc6 and MCM proteins modulate their biochemical properties *Nucleic Acids Res*. 33:4940–50 [PMID: 16150924][DOI]

102. Samson RY, Abeyrathne PD & Bell SD (2016) Mechanism of Archaeal MCM Helicase Recruitment to DNA Replication Origins *Mol*. *Cell* 61:287–96 [PMID: 26725007][DOI]

103. Dueber EC, Costa A, Corn JE, Bell SD & Berger JM (2011) Molecular determinants of origin discrimination by Orc1 initiators in archaea *Nucleic Acids Res*. 39:3621–31 [PMID: 21227921][DOI]

104. Matsunaga F, Takemura K, Akita M, Adachi A, Yamagami T & Ishino Y (2010) Localized melting of duplex DNA by Cdc6/Orc1 at the DNA replication origin in the hyperthermophilic archaeon Pyrococcus furiosus *Extremophiles* 14:21–31 [PMID: 19787415][DOI]

105. Onishi M, Liou GG, Buchberger JR, Walz T & Moazed D (2007) Role of the conserved Sir3-BAH domain in nucleosome binding and silent chromatin assembly *Mol*. *Cell* 28:1015–28 [PMID: 18158899][DOI]

106. Kuo AJ, Song J, Cheung P, Ishibe-Murakami S, Yamazoe S, Chen JK, Patel DJ & Gozani O (2012) The BAH domain of ORC1 links H4K20me2 to DNA replication licensing and Meier-Gorlin syndrome *Nature* 484:115–9 [PMID: 22398447][DOI]

107. Bleichert F, Botchan MR & Berger JM (2017) Mechanisms for initiating cellular DNA replication *Science* 355:aah6317 [PMID: 28209641][DOI]

108. Gambus A, Khoudoli GA, Jones RC & Blow JJ (2011) MCM2-7 form double hexamers at licensed origins in Xenopus egg extract *J*. *Biol*. *Chem*. 286:11855–64 [PMID: 21282109][DOI]

109. Remus D, Beuron F, Tolun G, Griffith JD, Morris EP & Diffley JF (2009) Concerted loading of Mcm2-7 double hexamers around DNA during DNA replication origin licensing *Cell* 139:719–30 [PMID: 19896182][DOI]

110. Evrin C, Clarke P, Zech J, Lurz R, Sun J, Uhle S, Li H, Stillman B & Speck C (2009) A double-hexameric MCM2-7 complex is loaded onto origin DNA during licensing of eukaryotic DNA replication *Proc*. *Natl*. *Acad*. *Sci*. *U*.*S*.*A*. 106:20240–5 [PMID: 19910535][DOI]

111. Ge XQ, Jackson DA & Blow JJ (2007) Dormant origins licensed by excess Mcm2-7 are required for human cells to survive replicative stress *Genes Dev*. 21:3331–41 [PMID: 18079179][DOI]

112. Ibarra A, Schwob E & Méndez J (2008) Excess MCM proteins protect human cells from replicative stress by licensing backup origins of replication *Proc*. *Natl*. *Acad*. *Sci*. *U*.*S*.*A*. 105:8956–61 [PMID: 18579778][DOI]

113. Stinchcomb DT, Struhl K & Davis RW (1979) Isolation and characterisation of a yeast chromosomal replicator *Nature* 282:39–43 [PMID: 388229]

114. Huberman JA, Spotila LD, Nawotka KA, el-Assouli SM & Davis LR (1987) The in vivo replication origin of the yeast 2 microns plasmid *Cell* 51:473–81 [PMID: 3311385]

115. Brewer BJ & Fangman WL (1987) The localization of replication origins on ARS plasmids in S. cerevisiae *Cell* 51:463–71 [PMID: 2822257]

116. Marahrens Y & Stillman B (1992) A yeast chromosomal origin of DNA replication defined by multiple functional elements *Science* 255:817–23 [PMID: 1536007]

117. Rao H, Marahrens Y & Stillman B (1994) Functional conservation of multiple elements in yeast chromosomal replicators *Mol*. *Cell*. *Biol*.14:7643–51 [PMID: 7935478]

118. Broach JR, Li YY, Feldman J, Jayaram M, Abraham J, Nasmyth KA & Hicks JB (1983) Localization and sequence analysis of yeast origins of DNA replication *Cold Spring Harb*. *Symp*. *Quant*. *Biol*. 47 Pt 2:1165–73 [PMID: 6345070]

119. Celniker SE, Sweder K, Srienc F, Bailey JE & Campbell JL (1984) Deletion mutations affecting autonomously replicating sequence ARS1 of Saccharomyces cerevisiae *Mol*. *Cell*. *Biol*. 4:2455–66 [PMID: 6392851]

120. Rao H & Stillman B (1995) The origin recognition complex interacts with a bipartite DNA binding site within yeast replicators *Proc*. *Natl*. *Acad*. *Sci*. *U*.*S*.*A*. 92:2224–8 [PMID: 7892251]

121. Rowley A, Cocker JH, Harwood J & Diffley JF (1995) Initiation complex assembly at budding yeast replication origins begins with the recognition of a bipartite sequence by limiting amounts of the initiator, ORC *EMBO J*. 14:2631–41 [PMID: 7781615]

122. Bell SP & Stillman B (1992) ATP-dependent recognition of eukaryotic origins of DNA replication by a multiprotein complex *Nature* 357:128–34 [PMID: 1579162][DOI]

123. Li N, Lam WH, Zhai Y, Cheng J, Cheng E, Zhao Y, Gao N & Tye BK (2018) Structure of the origin recognition complex bound to DNA replication origin *Nature* 559:217–22 [PMID: 29973722][DOI]

124. Bleichert F, Botchan MR & Berger JM (2015) Crystal structure of the eukaryotic origin recognition complex *Nature* 519:321–6 [PMID: 25762138][DOI]

125. Sun J, Evrin C, Samel SA, Fernández-Cid A, Riera A, Kawakami H, Stillman B, Speck C & Li H (2013) Cryo-EM structure of a helicase loading intermediate containing ORC-Cdc6-Cdt1-MCM2-7 bound to DNA *Nat*. *Struct*. *Mol*. *Biol*. 20:944–51 [PMID: 23851460][DOI]

126. Kawakami H, Ohashi E, Kanamoto S, Tsurimoto T & Katayama T (2015) Specific binding of eukaryotic ORC to DNA replication origins depends on highly conserved basic residues *Sci*. *Rep*. 5:14929 [PMID: 26456755][DOI]

127. Palzkill TG & Newlon CS (1988) A yeast replication origin consists of multiple copies of a small conserved sequence *Cell* 53:441–50 [PMID: 3284655]

128. Wilmes GM & Bell SP (2002) The B2 element of the Saccharomyces cerevisiae ARS1 origin of replication requires specific sequences to facilitate pre-RC formation *Proc*. *Natl*. *Acad*. *Sci*. *U*.*S*.*A*. 99:101–6 [PMID: 11756674][DOI]

129. Coster G & Diffley JFX (2017) Bidirectional eukaryotic DNA replication is established by quasi-symmetrical helicase loading *Science* 357:314–8 [PMID: 28729513][DOI]

130. Zou L & Stillman B (2000) Assembly of a complex containing Cdc45p, replication protein A, and Mcm2p at replication origins controlled by S-phase cyclin-dependent kinases and Cdc7p-Dbf4p kinase *Mol*. *Cell*. *Biol*. 20:3086–96 [PMID: 10757793]

131. Lipford JR & Bell SP (2001) Nucleosomes positioned by ORC facilitate the initiation of DNA replication *Mol*. *Cell* 7:21–30 [PMID: 11172708]

132. Diffley JF & Cocker JH (1992) Protein-DNA interactions at a yeast replication origin *Nature* 357:169–72 [PMID: 1579168][DOI]

133. Diffley JF & Stillman B (1988) Purification of a yeast protein that binds to origins of DNA replication and a transcriptional silencer *Proc*. *Natl*. *Acad*. *Sci*. *U*.*S*.*A*. 85:2120–4 [PMID: 3281162]

134. Miotto B, Ji Z & Struhl K (2016) Selectivity of ORC binding sites and the relation to replication timing, fragile sites, and deletions in cancers *Proc*. *Natl*. *Acad*. *Sci*. *U*.*S*.*A*. 113:E4810-9 [PMID: 27436900][DOI]

135. MacAlpine HK, Gordân R, Powell SK, Hartemink AJ & MacAlpine DM (2010) Drosophila ORC localizes to open chromatin and marks sites of cohesin complex loading *Genome Res*. 20:201–11 [PMID: 19996087][DOI]

136. Eaton ML, Prinz JA, MacAlpine HK, Tretyakov G, Kharchenko PV & MacAlpine DM (2011) Chromatin signatures of the Drosophila replication program *Genome Res*. 21:164–74 [PMID: 21177973][DOI]

137. Dellino GI, Cittaro D, Piccioni R, Luzi L, Banfi S, Segalla S, Cesaroni M, Mendoza-Maldonado R, Giacca M & Pelicci PG (2013) Genome-wide mapping of human DNA-replication origins: levels of transcription at ORC1 sites regulate origin selection and replication timing *Genome Res*. 23:1–11 [PMID: 23187890][DOI]

138. Cayrou C, Ballester B, Peiffer I, Fenouil R, Coulombe P, Andrau JC, van Helden J & Méchali M (2015) The chromatin environment shapes DNA replication origin organization and defines origin classes *Genome Res*. 25:1873–85 [PMID: 26560631][DOI]

139. Cayrou C, Coulombe P, Vigneron A, Stanojcic S, Ganier O, Peiffer I, Rivals E, Puy A, Laurent-Chabalier S, Desprat R & Méchali M (2011) Genome-scale analysis of metazoan replication origins reveals their organization in specific but flexible sites defined by conserved features *Genome Res*. 21:1438–49 [PMID: 21750104][DOI]

140. Lubelsky Y, Sasaki T, Kuipers MA, Lucas I, Le Beau MM, Carignon S, Debatisse M, Prinz JA, Dennis JH & Gilbert DM (2011) Pre-replication complex proteins assemble at regions of low nucleosome occupancy within the Chinese hamster dihydrofolate reductase initiation zone *Nucleic Acids Res*. 39:3141–55 [PMID: 21148149][DOI]

141. Hayashi M, Katou Y, Itoh T, Tazumi A, Tazumi M, Yamada Y, Takahashi T, Nakagawa T, Shirahige K & Masukata H (2007) Genome-wide localization of pre-RC sites and identification of replication origins in fission yeast *EMBO J*. 26:1327–39 [PMID: 17304213][DOI]

142. Martin MM, Ryan M, Kim R, Zakas AL, Fu H, Lin CM, Reinhold WC, Davis SR, Bilke S, Liu H, Doroshow JH, Reimers MA, Valenzuela MS, Pommier Y, Meltzer PS & Aladjem MI (2011) Genome-wide depletion of replication initiation events in highly transcribed regions *Genome Res*. 21:1822–32 [PMID: 21813623][DOI]

143. Pourkarimi E, Bellush JM & Whitehouse I (2016) Spatiotemporal coupling and decoupling of gene transcription with DNA replication origins during embryogenesis in *C*. *elegans eLife* 5:e21728 [PMID: 28009254][DOI]

144. Rodríguez-Martínez M, Pinzón N, Ghommidh C, Beyne E, Seitz H, Cayrou C & Méchali M (2017) The gastrula transition reorganizes replication-origin selection in Caenorhabditis elegans *Nat*. *Struct*. *Mol*. *Biol*. 24:290–9 [PMID: 28112731][DOI]

145. Besnard E, Babled A, Lapasset L, Milhavet O, Parrinello H, Dantec C, Marin JM & Lemaitre JM (2012) Unraveling cell type-specific and reprogrammable human replication origin signatures associated with G-quadruplex consensus motifs *Nat*. *Struct*. *Mol*. *Biol*. 19:837–44 [PMID: 22751019][DOI]

146. Delgado S, Gómez M, Bird A & Antequera F (1998) Initiation of DNA replication at CpG islands in mammalian chromosomes *EMBO J*. 17:2426–35 [PMID: 9545253][DOI]

147. Sequeira-Mendes J, Díaz-Uriarte R, Apedaile A, Huntley D, Brockdorff N & Gómez M (2009) Transcription initiation activity sets replication origin efficiency in mammalian cells *PLoS Genet*. 5:e1000446 [PMID: 19360092][DOI]

148. Kelly T & Callegari AJ (2019) Dynamics of DNA replication in a eukaryotic cell *Proc*. *Natl*. *Acad*. *Sci*. *U*.*S*.*A*. 116:4973–82 [PMID: 30718387][DOI]

149. Austin RJ, Orr-Weaver TL & Bell SP (1999) Drosophila ORC specifically binds to ACE3, an origin of DNA replication control element *Genes Dev*. 13:2639–49 [PMID: 10541550][DOI]

150. Beall EL, Manak JR, Zhou S, Bell M, Lipsick JS & Botchan MR (2002) Role for a Drosophila Myb-containing protein complex in site-specific DNA replication *Nature* 420:833–7 [PMID: 12490953][DOI]

151. Beall EL, Bell M, Georlette D & Botchan MR (2004) Dm-myb mutant lethality in Drosophila is dependent upon mip130: positive and negative regulation of DNA replication *Genes Dev*. 18:1667–80 [PMID: 15256498][DOI]

152. Lewis PW, Beall EL, Fleischer TC, Georlette D, Link AJ & Botchan MR (2004) Identification of a Drosophila Myb-E2F2/RBF transcriptional repressor complex *Genes Dev*. 18:2929–40 [PMID: 15545624][DOI]

153. Bosco G, Du W & Orr-Weaver TL (2001) DNA replication control through interaction of E2F-RB and the origin recognition complex *Nat*. *Cell Biol*. 3:289–95 [PMID: 11231579][DOI]

154. Chuang RY & Kelly TJ (1999) The fission yeast homologue of Orc4p binds to replication origin DNA via multiple AT-hooks *Proc*. *Natl*. *Acad*. *Sci*. *U*.*S*.*A*. 96:2656–61 [PMID: 10077566]

155. Balasov M, Huijbregts RP & Chesnokov I (2007) Role of the Orc6 protein in origin recognition complex-dependent DNA binding and replication in Drosophila melanogaster *Mol*. *Cell*. *Biol*. 27:3143–53 [PMID: 17283052][DOI]

156. Tardat M, Brustel J, Kirsh O, Lefevbre C, Callanan M, Sardet C & Julien E (2010) The histone H4 Lys 20 methyltransferase PR-Set7 regulates replication origins in mammalian cells *Nat*. *Cell Biol*. 12:1086–93 [PMID: 20953199][DOI]

157. Beck DB, Burton A, Oda H, Ziegler-Birling C, Torres-Padilla ME & Reinberg D (2012) The role of PR-Set7 in replication licensing depends on Suv4-20h *Genes Dev*. 26:2580–9 [PMID: 23152447][DOI]

158. Brustel J, Kirstein N, Izard F, Grimaud C, Prorok P, Cayrou C, Schotta G, Abdelsamie AF, Déjardin J, Méchali M, Baldacci G, Sardet C, Cadoret JC, Schepers A & Julien E (2017) Histone H4K20 tri-methylation at late-firing origins ensures timely heterochromatin replication *EMBO J*. 36:2726–41 [PMID: 28778956][DOI]

159. Shoaib M, Walter D, Gillespie PJ, Izard F, Fahrenkrog B, Lleres D, Lerdrup M, Johansen JV, Hansen K, Julien E, Blow JJ & Sørensen CS (2018) Histone H4K20 methylation mediated chromatin compaction threshold ensures genome integrity by limiting DNA replication licensing *Nat*. *Commun*. 9:3704 [PMID: 30209253][DOI]

160. Noguchi K, Vassilev A, Ghosh S, Yates JL & DePamphilis ML (2006) The BAH domain facilitates the ability of human Orc1 protein to activate replication origins in vivo *EMBO J*. 25:5372–82 [PMID: 17066079][DOI]

161. Shen Z, Chakraborty A, Jain A, Giri S, Ha T, Prasanth KV & Prasanth SG (2012) Dynamic association of ORCA with prereplicative complex components regulates DNA replication initiation *Mol*. *Cell*. *Biol*. 32:3107–20 [PMID: 22645314][DOI]

162. Wang Y, Khan A, Marks AB, Smith OK, Giri S, Lin YC, Creager R, MacAlpine DM, Prasanth KV, Aladjem MI & Prasanth SG (2017) Temporal association of ORCA/LRWD1 to late-firing origins during G1 dictates heterochromatin replication and organization *Nucleic Acids Res*. 45:2490–502 [PMID: 27924004][DOI]

163. Bartke T, Vermeulen M, Xhemalce B, Robson SC, Mann M & Kouzarides T (2010) Nucleosome-interacting proteins regulated by DNA and histone methylation *Cell* 143:470–84 [PMID: 21029866][DOI]

164. Vermeulen M, Eberl HC, Matarese F, Marks H, Denissov S, Butter F, Lee KK, Olsen JV, Hyman AA, Stunnenberg HG & Mann M (2010) Quantitative interaction proteomics and genome-wide profiling of epigenetic histone marks and their readers *Cell* 142:967–80 [PMID: 20850016][DOI]

165. Hein MY, Hubner NC, Poser I, Cox J, Nagaraj N, Toyoda Y, Gak IA, Weisswange I, Mansfeld J, Buchholz F, Hyman AA & Mann M (2015) A human interactome in three quantitative dimensions organized by stoichiometries and abundances *Cell* 163:712–23 [PMID: 26496610][DOI]

166. Thomae AW, Pich D, Brocher J, Spindler MP, Berens C, Hock R, Hammerschmidt W & Schepers A (2008) Interaction between HMGA1a and the origin recognition complex creates site-specific replication origins *Proc*. *Natl*. *Acad*. *Sci*. *U*.*S*.*A*. 105:1692–7 [PMID: 18234858][DOI]

167. Zhang Y, Huang L, Fu H, Smith OK, Lin CM, Utani K, Rao M, Reinhold WC, Redon CE, Ryan M, Kim R, You Y, Hanna H, Boisclair Y, Long Q & Aladjem MI (2016) A replicator-specific binding protein essential for site-specific initiation of DNA replication in mammalian cells *Nat*. *Commun*. 7:11748 [PMID: 27272143][DOI]

168. Bleichert F, Leitner A, Aebersold R, Botchan MR & Berger JM (2018) Conformational control and DNA-binding mechanism of the metazoan origin recognition complex *Proc*. *Natl*. *Acad*. *Sci*. *U*.*S*.*A*. 115:E5906-15 [PMID: 29899147][DOI]

169. Clarey MG, Botchan M & Nogales E (2008) Single particle EM studies of the Drosophila melanogaster origin recognition complex and evidence for DNA wrapping *J*. *Struct*. *Biol*. 164:241–9 [PMID: 18824234][DOI]

170. Lee DG & Bell SP (1997) Architecture of the yeast origin recognition complex bound to origins of DNA replication *Mol*. *Cell*. *Biol*. 17:7159–68 [PMID: 9372948]

171. Riera A, Barbon M, Noguchi Y, Reuter LM, Schneider S & Speck C (2017) From structure to mechanism-understanding initiation of DNA replication *Genes Dev*. 31:1073–88 [PMID: 28717046][DOI]

172. Tognetti S, Riera A & Speck C (2015) Switch on the engine: how the eukaryotic replicative helicase MCM2-7 becomes activated *Chromosoma* 124:13–26 [PMID: 25308420][DOI]

173. Berbenetz NM, Nislow C & Brown GW (2010) Diversity of eukaryotic DNA replication origins revealed by genome-wide analysis of chromatin structure *PLoS Genet*. 6:e1001092 [PMID: 20824081][DOI]

174. Eaton ML, Galani K, Kang S, Bell SP & MacAlpine DM (2010) Conserved nucleosome positioning defines replication origins *Genes Dev*. 24:748–53 [PMID: 20351051][DOI]

175. Azmi IF, Watanabe S, Maloney MF, Kang S, Belsky JA, MacAlpine DM, Peterson CL & Bell SP (2017) Nucleosomes influence multiple steps during replication initiation *eLife* 6:e22512 [PMID: 28322723][DOI]

176. Miotto B & Struhl K (2010) HBO1 histone acetylase activity is essential for DNA replication licensing and inhibited by Geminin *Mol*. *Cell* 37:57–66 [PMID: 20129055][DOI]

177. Liu J, Zimmer K, Rusch DB, Paranjape N, Podicheti R, Tang H & Calvi BR (2015) DNA sequence templates adjacent nucleosome and ORC sites at gene amplification origins in Drosophila *Nucleic Acids Res*. 43:8746–61 [PMID: 26227968][DOI]

178. Zhao PA, Rivera-Mulia JC & Gilbert DM (2017) Replication Domains: Genome Compartmentalization into Functional Replication Units *Adv*. *Exp*. *Med*. *Biol*. 1042:229–57 [PMID: 29357061][DOI]

179. Sugimoto N & Fujita M (2017) Molecular Mechanism for Chromatin Regulation During MCM Loading in Mammalian Cells *Adv*. *Exp*. *Med*. *Biol*. 1042:61–78 [PMID: 29357053][DOI]

180. MacAlpine DM & Almouzni G (2013) Chromatin and DNA replication *Cold Spring Harb*. *Perspect*. *Biol*. 5:a010207 [PMID: 23751185][DOI]

181. Sima J, Chakraborty A, Dileep V, Michalski M, Klein KN, Holcomb NP, Turner JL, Paulsen MT, Rivera-Mulia JC, Trevilla-Garcia C, Bartlett DA, Zhao PA, Washburn BK, Nora EP, Kraft K, Mundlos S, Bruneau BG, Ljungman M, Fraser P, Ay F & Gilbert DM (2019) Identifying cis Elements for Spatiotemporal Control of Mammalian DNA Replication *Cell* 176:816–30 [PMID: 30595451][DOI]

182. Cadoret JC, Meisch F, Hassan-Zadeh V, Luyten I, Guillet C, Duret L, Quesneville H & Prioleau MN (2008) Genome-wide studies highlight indirect links between human replication origins and gene regulation *Proc*. *Natl*. *Acad*. *Sci*. *U*.*S*.*A*. 105:15837–42 [PMID: 18838675][DOI]

183. Gros J, Kumar C, Lynch G, Yadav T, Whitehouse I & Remus D (2015) Post-licensing Specification of Eukaryotic Replication Origins by Facilitated Mcm2-7 Sliding along DNA *Mol*. *Cell* 60:797–807 [PMID: 26656162][DOI]

184. Letessier A, Millot GA, Koundrioukoff S, Lachagès AM, Vogt N, Hansen RS, Malfoy B, Brison O & Debatisse M (2011) Cell-type-specific replication initiation programs set fragility of the FRA3B fragile site *Nature* 470:120–3 [PMID: 21258320][DOI]

185. Smith OK, Kim R, Fu H, Martin MM, Lin CM, Utani K, Zhang Y, Marks AB, Lalande M, Chamberlain S, Libbrecht MW, Bouhassira EE, Ryan MC, Noble WS & Aladjem MI (2016) Distinct epigenetic features of differentiation-regulated replication origins *Epigenetics Chromatin* 9:18 [PMID: 27168766][DOI]

186. Sher N, Bell GW, Li S, Nordman J, Eng T, Eaton ML, Macalpine DM & Orr-Weaver TL (2012) Developmental control of gene copy number by repression of replication initiation and fork progression *Genome Res*. 22:64–75 [PMID: 22090375][DOI]

187. Comoglio F, Schlumpf T, Schmid V, Rohs R, Beisel C & Paro R (2015) High-resolution profiling of Drosophila replication start sites reveals a DNA shape and chromatin signature of metazoan origins *Cell Rep*. 11:821–34 [PMID: 25921534][DOI]

188. Calvi BR, Lilly MA & Spradling AC (1998) Cell cycle control of chorion gene amplification *Genes Dev*. 12:734–44 [PMID: 9499407]

189. Mosig G (1998) Recombination and recombination-dependent DNA replication in bacteriophage T4 *Annu*. *Rev*. *Genet*. 32:379–413 [PMID: 9928485][DOI]

190. Ravoitytė B & Wellinger RE (2017) Non-Canonical Replication Initiation: You're Fired! *Genes (Basel)* 8:E54 [PMID: 28134821][DOI]

191. Asai T, Sommer S, Bailone A & Kogoma T (1993) Homologous recombination-dependent initiation of DNA replication from DNA damage-inducible origins in Escherichia coli *EMBO J*. 12:3287–95 [PMID: 8344265]

192. Lydeard JR, Jain S, Yamaguchi M & Haber JE (2007) Break-induced replication and telomerase-independent telomere maintenance require Pol32 *Nature* 448:820–3 [PMID: 17671506][DOI]

193. Dasgupta S, Masukata H & Tomizawa J (1987) Multiple mechanisms for initiation of ColE1 DNA replication: DNA synthesis in the presence and absence of ribonuclease H *Cell* 51:1113–22 [PMID: 2446774]

194. Stuckey R, García-Rodríguez N, Aguilera A & Wellinger RE (2015) Role for RNA:DNA hybrids in origin-independent replication priming in a eukaryotic system *Proc*. *Natl*. *Acad*. *Sci*. *U*.*S*.*A*. 112:5779–84 [PMID: 25902524][DOI]

195. Burki F (2014) The eukaryotic tree of life from a global phylogenomic perspective *Cold Spring Harb*. *Perspect*. *Biol*. 6:a016147 [PMID: 24789819][DOI]

196. Lee PH, Meng X & Kapler GM (2015) Developmental regulation of the Tetrahymena thermophila origin recognition complex *PLoS Genet*. 11:e1004875 [PMID: 25569357][DOI]

197. Mohammad MM, Donti TR, Sebastian Yakisich J, Smith AG & Kapler GM (2007) Tetrahymena ORC contains a ribosomal RNA fragment that participates in rDNA origin recognition *EMBO J*. 26:5048–60 [PMID: 18007594][DOI]

198. Donti TR, Datta S, Sandoval PY & Kapler GM (2009) Differential targeting of Tetrahymena ORC to ribosomal DNA and non-rDNA replication origins *EMBO J*. 28:223–33 [PMID: 19153611][DOI]

199. Marques CA & McCulloch R (2018) Conservation and Variation in Strategies for DNA Replication of Kinetoplastid Nuclear Genomes *Curr*. *Genomics* 19:98–109 [PMID: 29491738][DOI]

200. Marques CA, Tiengwe C, Lemgruber L, Damasceno JD, Scott A, Paape D, Marcello L & McCulloch R (2016) Diverged composition and regulation of the Trypanosoma brucei origin recognition complex that mediates DNA replication initiation *Nucleic Acids Res*. 44:4763–84 [PMID: 26951375][DOI]

201. Tiengwe C, Marcello L, Farr H, Gadelha C, Burchmore R, Barry JD, Bell SD & McCulloch R (2012) Identification of ORC1/CDC6-interacting factors in Trypanosoma brucei reveals critical features of origin recognition complex architecture *PLoS ONE* 7:e32674 [PMID: 22412905][DOI]

202. Marques CA, Dickens NJ, Paape D, Campbell SJ & McCulloch R (2015) Genome-wide mapping reveals single-origin chromosome replication in Leishmania, a eukaryotic microbe *Genome Biol*. 16:230 [PMID: 26481451][DOI]
